# Early risk markers for severe clinical course and fatal outcome in German patients with COVID-19

**DOI:** 10.1371/journal.pone.0246182

**Published:** 2021-01-29

**Authors:** Paul Balfanz, Bojan Hartmann, Dirk Müller-Wieland, Michael Kleines, Dennis Häckl, Nils Kossack, Alexander Kersten, Christian Cornelissen, Tobias Müller, Ayham Daher, Robert Stöhr, Johannes Bickenbach, Gernot Marx, Nikolaus Marx, Michael Dreher

**Affiliations:** 1 Department of Cardiology, Angiology and Intensive Care Medicine, University Hospital Aachen, Aachen, Germany; 2 Department of Medical Microbiology, University Hospital Aachen, Aachen, Germany; 3 WIG2 –Scientific Institute for Health Economics and Health System Research, Leipzig, Germany; 4 Department of Pneumology and Intensive Care Medicine, University Hospital Aachen, Aachen, Germany; 5 Department of Surgical Intensive Medicine and Intermediate Care, University Hospital Aachen, Aachen, Germany; University of Mississippi Medical Center, UNITED STATES

## Abstract

**Background:**

Some patients with Corona Virus Disease 2019 (COVID-19) develop a severe clinical course with acute respiratory distress syndrome (ARDS) and fatal outcome. Clinical manifestations and biomarkers in early stages of disease with relevant predictive impact for outcomes remain largely unexplored. We aimed to identify parameters which are significantly different between subgroups.

**Design:**

125 patients with COVID-19 were analysed. Patients with ARDS (N = 59) or non-ARDS (N = 66) were compared, as well as fatal outcome versus survival in the two groups.

**Key results:**

ARDS and non-ARDS patients did not differ with respect to comorbidities or medication on developing a fatal outcome versus survival. Body mass index was higher in patients with ARDS versus non-ARDS (p = 0.01), but not different within the groups in survivors versus non-survivors. Interleukin-6 levels on admission were higher in patients with ARDS compared to non-ARDS as well as in patients with fatal outcome versus survivors, whereas lymphocyte levels were lower in the different subgroups (all p<0.05). There was a highly significant 3.5-fold difference in fever load in non-survivors compared to survivors (p<0.0001). Extrapulmonary viral spread was detected more often in patients with fatal outcome compared to survivors (P = 0.01). Further the detection of SARS-CoV-2 in serum showed a significantly more severe course and an increased risk of death (both p<0.05).

**Conclusions:**

We have identified early risk markers for a severe clinical course, like ARDS or fatal outcome. This data might help develop a strategy to address new therapeutic options early in patients with COVID-19 and at high risk for fatal outcome.

## Introduction

Recently, a new type of Coronavirus, SARS-CoV (Severe Acute Respiratory Syndrome Corona Virus)-2, led to a worldwide pandemic outbreak of an infectious disease, called COVID-19 (Corona Virus Disease 2019). The clinical manifestation of this disease is very broad and variable, ranging from asymptomatic carriers to symptoms of acute infection of the upper airways and occasionally severe acute respiratory insufficiency and death [1–3]. Various risk factors and comorbidities potentially modulating susceptibility to infection and severity of disease are discussed, but it is not clear which factors determine not only the clinical course, but also the fate of patients with COVID-19 [4].

Although COVID-19 appears to have a lower fatality rate than infections with SARS-CoV or Middle East Respiratory Syndrome (MERS)-CoV, the absolute number of deaths is high due to the global burden of infection. Beside possible regional differences in health care, an age-related increase in mortality has consistently been observed. Recently, based on results from an observational database of 169 hospitals in Asia, Europe, and North America, cardiovascular and pulmonary comorbidities have been reported to be independently associated with increased in-hospital death [4]. Furthermore, a decrease in kidney function and need for mechanical ventilation have been described as prognostic factors for fatal outcome in 5,700 patients hospitalized with COVID-19 in the New York City area [5]. Next to increasing age, mechanical ventilation and higher PEEP level requirements, were associated with increased mortality in 1,591 COVID-19 patients admitted to the ICU departments in the Lombardy Region in Italy [6]. A large retrospective cohort study from Wuhan in China proposed older age, a high SOFA score, and D-Dimer levels greater than 1 μg/mL, as markers to identify poor prognosis [7].

However, none of these studies focus on predictors of severe clinical course and fatal outcome soon after hospital admission. In addition to being clinically relevant, such predictors are crucial for early identification of high-risk individuals, as these patients may benefit from early novel treatment strategies.

In a preliminary report, we presented clinical data from 50 patients hospitalized due to COVID-19 [8]. In the present study, we retrospectively evaluated 125 COVID-19 patients admitted to the University Hospital in Aachen, Germany. We compared patients with fatal outcome versus survivors with a disease severity of ARDS or non-ARDS, and propose early clinical markers that may help predict fatal outcome.

## Methods

For the clinical description of the first 125 patients admitted with COVID-19 consecutively, we retrospectively evaluated data from all patients admitted to the University Hospital in Aachen, Germany, at the start of the SARS-CoV-2 pandemic on February 24^th^ 2020, until July 30^th^. Observation of the first 50 patients has been described initially [8]. A diagnosis was made based on a positive SARS-CoV-2 result in respiratory samples in our hospital, externally before admission, or transferred from another hospital. Patients were either isolated under standard care or treated in our intensive care unit. The different treatment strategies and consequently the group definition was defined by severity of the disease. Severity of ARDS was classified according to the degree of hypoxia as defined by the “Berlin definition”. Patients with ARDS were treated in our intensive care units. Patients without ARDS not needing intensive care medicine were isolated under standard care. To identify potential predictors of clinical outcome in COVID-19 patients, we focused on the analysis of various parameters in non-survivors and survivors. Survivors were discharged from the hospital after treatment, whereas non-survivors died in connection to COVID-19 disease.

Comorbidities (such as hypertension, overweight or obesity, pre-existing respiratory or cardiovascular diseases, smoking, chronic kidney disease, malignancies, chronic liver disease), and medications prescribed at the time of admission were recorded in hospital, or taken from existing medical records. We evaluated early symptoms, as well as timing of initial physician contact and hospitalization.

A body mass index (BMI) of 25 to < 30 kg/m^2^ was classified as overweight, and obesity as ≥ 30 kg/m^2^. Diabetes or prediabetes was defined by clinical history, medication and HbA1c values ≥ 6.5%, or ≥ 5.7 to < 6.5%, respectively.

Febrile days were defined as the time from fever onset until the last documented value above 38.5°C.

Vital parameters presented in this study were taken between four and 24 hours following hospital admission or intubation, with the worst values being depicted. Severity of ARDS was defined using P/F-ratio, or the Horowitz index: an index below 100 mmHg defines severe ARDS, below 200 mmHg moderate ARDS, and below 300 mmHg mild ARDS.

Diagnostics of viral infection was performed by broncho alveolar lavage (BAL) in each intubated patient. In spontanous breathing patients, sputum was used for testing. Viral load was determined by real-time (rt)-polymerase chain reaction (PCR) of the sample. The threshold value Ct represents the time point, at which the exponential phase of amplification begins, which therefore is inversely proportional to the virus concentration in the material and reflects the relative difference in a logarithmic scale. The threshold value of the sample gene < 20 was classified as high. Values > 30 were classified as low viral load, and values of 20 to < 30 were medium. The same applies when serum, urine or stool were analysed for the presence of the SARS-CoV-2 virus.

The blood tests after hospital admission were also analysed for white blood count and lymphopenia; the latter was diagnosed with relative lymphocyte counts below 22% using flow cytometry and 25% using microscopic analysis, or with an absolute lymphocyte count below 1,0/nL. Further blood tests were analysed regularly as indicated, therefore patient numbers vary between different time points in the figures, but the time point refers to the initial admission for each patient.

Further technical and imagery tests were performed based on clinical decision making and evaluated in a standardized manner.

All parameters were tested for significance as described in the legends for all tables and figures. Nominal scaled parameters testing according to Fisher was performed, whereas ordinal scaled variables were tested for normal distribution and the Welsh test was used. Otherwise the Wilcoxon-Mann-Whitney-test was used. Categorial variables were tested by Pearson’s chi-squared test. Data are presented as mean ± standard error of the mean or median values with interquartile range (IQR) or confidence interval of 95%. Statistical significance was assumed for a p-value of < 0.05. Statistical testing was performed with GraphPad Prism version 8.4.3 and R version 3.6.3, utilizing packages ggplot2 (3.3.2) for plots, tangram (0.7.1) for summary statistics, base R generalized linear models (glm) for logistic regression and etm (1.1.1) for estimating cumulative incidence functions.

The event of intubation in days after symptom onset of all ARDS patients was estimated by the Kaplan-Meier method and described for the specific outcome.

The study obtained an ethics approval from the ethics committee at the RWTH Aachen Faculty of Medicine. All data were fully anonymized and patients provided informed written consent.

## Results

This cohort summarizes the first 125 COVID-19 patients in the University Hospital of Aachen. Aachen was an epicenter of the disease in Germany, and is located close to Heinsberg, the area in which the first serious outbreak was detected in Germany. 59 patients with ARDS were treated in the intensive care unit, while 66 patients were admitted to a regular isolation ward. At the time of this analysis, 38 of the 125 patients were deceased (30%), and 87 (70%) had been discharged from hospital.

## Patient characteristics

Baseline characteristics of the overall cohort and subgroups with ARDS and non-ARDS and for the subgroups of non-survivors versus survivors are summarized in Table 1. In the overall cohort, mean age was 66±1.2 years, and 30% were women. In the subgroups of ARDS and non-ARDS patients survivors were younger compared to patients with fatal outcome (ARDS: 63.1±3.1 versus 66.2±4 years; p = 0.1; non-ARDS: 65.4±4.6 versus 78.8±5.7; p<0.01) (Table 2). Main initial clinical findings included fever (72%), dyspnea and cough (55% each), and one third of patients reported gastrointestinal symptoms. In the total population, the time from onset of first clinical symptoms to hospitalization was 5.0±0.5 days. The time from symptom onset to hospitalization was lower in patients with fatal outcome compared to survivors showing a significant difference in the subgroup of non ARDS patients (5.5±1.5 vs. 2.4±2.4; p = 0.04) (Table 2). Admission of ARDS patients on intensive care unit after symptom onset was 9.0±0.9 days, they were intubated after 10.0±1.0 days after symptom onset. All patients had comorbiditie, but in the performed univariate and multivariate logistic regression analysis there were no highly significant differences in prevalence of arterial hypertension, pre-existing respiratory diseases, pre-existing heart diseases or medications between patients with ARDS compared to non-ARDS patients and between patients with fatal outcome compared to survivors (Table 1).

**Table 1:** Baseline characteristics of the different subgroups.

	N (%)				N (%)		
	Total (N = 125)	ARDS patients (N = 59)	Non-ARDS patients (N = 66)	P values	Non-Survivors (N = 38)	Survivors (N = 87)	P values
**Subgroups**	** **	** **	** **	** **	** **	** **	** **
ARDS patients	-	-	-	**-**	26 (44)	33 (56)	**0.001**
Non-ARDS patients	-	-	-	**-**	12 (18)	54 (82)	
**Characteristics**							
Age—Mean (±SEM), years	66 (±1.2)	64 (±1.2)	68 (±2)	**0.01**	70 (±1.8)	65 (±1.5)	**0.03**
Female sex—N (%)	38 (30)	21 (36)	17 (26)	0.24	12 (32)	26 (30)	0.83
**Comorbidities**							
Total	120 (96)	57 (97)	63 (95)	>0.99	37 (97)	83 (95)	>0.99
Arterial hypertension	90 (72)	42 (71)	48 (73)	>0.99	32 (84)	58 (67)	0.05
Pre-existing respiratory disease	55 (44)	24 (41)	31 (47)	0.58	17 (45)	37 (43)	0.84
Pre-existing heart diseases	49 (39)	22 (37)	27 (41)	0.71	19 (50)	30 (34)	0.57
Overweight (BMI ≥ 25 kg/m^2^, < 30 kg/m^2^)	42 (34)	22 (37)	25 (38)	>0.99	12 (32)	35 (40)	0.42
Obesity (BMI ≥ 30 kg/m^2^)	44 (35)	27 (46)	22 (33)	0.19	15 (39)	34 (39)	>0.99
Chronic kidney disease	36 (29)	13 (22)	23 (35)	0.16	12 (32)	24 (28)	0.52
Smoking	34 (27)	12 (20)	22 (33)	0.11	7 (18)	28 (32)	0.13
Former smoking	16 (13)	5 (8)	11 (17)	0.19	5 (13)	11 (13)	>0.99
Current smoking	18 (14)	7 (12)	11 (17)	0.61	2 (5)	17 (20)	0.05
Diabetes mellitus	31 (25)	12 (20)	19 (29)	0.30	13 (34)	19 (22)	0.18
Malignancy	31 (25)	11 (19)	20 (30)	0.15	10 (26)	21 (24)	0.82
Chronic liver failure	10 (8)	2 (3)	8 (12)	0.10	2 (5)	8 (9)	0.72
Chronic hepatitis	8 (6)	2 (3)	6 (9)	0.27	3 (8)	5 (6)	0.69
Peripheral aterial occlusive disease	8 (6)	4 (7)	4 (6)	>0.99	3 (8)	5 (6)	0.69
**Premedications**							
ACE-Inhibitors	31 (25)	10 (17)	21 (32)	0.06	9 (24)	22 (25)	>0.99
Angiotensin-receptor blockers	32 (26)	16 (27)	16 (24)	0.83	10 (26)	22 (25)	>0.99
Beta blocker	47 (38)	25 (42)	22 (33)	0.35	15 (39)	32 (37)	0.84
Calcium antagonists	34 (27)	12 (20)	22 (33)	0.11	6 (16)	28 (32)	0.07
Diuretics	50 (40)	18 (31)	32 (48)	**0.04**	15 (39)	35 (40)	>0.99
Antidiabetics	27 (22)	8 (14)	19 (29)	0.05	9 (24)	18 (21)	0.81
Lipid-lowering agents	41 (33)	21 (36)	20 (30)	0.57	16 (42)	25 (29)	0.15
Antiplatelets	38 (30)	19 (32)	19 (29)	0.70	11 (29)	27 (31)	>0.99
Anticoagulants	28 (22)	16 (27)	12 (18)	0.28	13 (34)	15 (17)	0.06
Inhaled bronchodilators	26 (21)	9 (15)	17 (26)	0.18	4 (11)	22 (25)	0.09
Inhaled glucocorticoids	22 (18)	10 (17)	12 (18)	>0.99	3 (8)	9 (10)	>0.99
Systemic glucocorticoids	8 (6)	3 (5)	5 (8)	0.72	3 (8)	5 (6)	0.69
Immunosuppressants	11 (9)	6 (10)	5 (8)	0.75	3 (8)	8 (9)	>0.99
NSAR	24 (19)	12 (20)	12 (18)	0.82	5 (13)	19 (22)	0.32
Antibiotics	36 (29)	26 (44)	10 (15)	**0.0006**	12 (32)	24 (28)	0.67
Virostatics	9 (7)	8 (14)	1 (2)	**0.01**	2 (5)	7 (8)	0.72
**Initial symptoms**							
Fever	90 (72)	45 (76)	45 (68)	0.32	27 (71)	63 (72)	>0.99
Dyspnea	69 (55)	40 (68)	29 (44)	**0.01**	23 (61)	46 (53)	0.44
Cough	69 (55)	30 (51)	39 (59)	0.37	22 (58)	47 (54)	0.70
Gastrointestinal symptoms	44 (35)	20 (34)	24 (36)	0.85	9 (24)	35 (40)	0.10
Diarrhea	34 (27)	17 (29)	17 (26)	0.84	7 (18)	27 (31)	0.19
Emesis	11 (9)	6 (10)	5 (8)	0.75	4 (11)	7 (8)	0.73
Nausea	20 (16)	9 (15)	11 (17)	>0.99	4 (11)	16 (18)	0.42
Fatigue	42 (34)	14 (24)	28 (42)	**0.03**	7 (18)	35 (40)	**0.02**
Tiredness	34 (27)	13 (22)	21 (32)	0.23	5 (13)	29 (33)	**0.02**
Myalgia	22 (18)	7 (12)	15 (23)	0.15	4 (11)	18 (21)	0.20
Loss of taste	19 (15)	5 (8)	14 (21)	0.07	2 (5)	17 (20)	0.05
Loss of smell	15 (12)	3 (5)	12 (18)	**0.02**	2 (5)	13 (15)	0.14
Sore throat	13 (10)	5 (8)	8 (12)	0.56	1 (3)	12 (14)	0.10
Headache	13 (10)	3 (5)	10 (15)	0.08	0 (0)	13 (15)	**0.009**
Angina pectoris	10 (8)	5 (8)	5 (8)	>0.99	1 (3)	9 (10)	0.28
Pharyngalgia	7 (6)	3 (5)	4 (6)	>0.99	0 (0)	7 (8)	0.10
Rhinorrhoea	4 (3)	2 (3)	2 (3)	>0.99	0 (0)	4 (5)	0.31

Data in N (%) or Mean (±SEM). In ordinal scaled parameters testing for normal distribution and calculation of P values with Welch test or Wilcoxon-Mann-Whitney test. P values of nominal scaled parameter calculated with Fisher’s exact test. SEM = standard error of the mean. ARDS = acute respiratory distress syndrome. BMI = body mass index. ACE = angiotensin-converting enzyme. NSAR = non-steroidal anti rheumatics.

**Table 2:** Fatal outcome and survival in ARDS and non ARDS patients.

	ARDS			non ARDS		
	Survivor (N = 33)	Non-Survivor (N = 26)	P value	Survivor (N = 54)	Non-Survivor (N = 12)	P value
**Characteristics**						
Age	63.1 [60.0–66.2]	66.2 [62.1–70.2]	0.10^2^	65.4 [60.8–70.0]	78.8 [73.1–84.4]	**<0.01**^2^
Female Sex	11 (33.3%)	10 (38.5%)	0.68^1^	15 (27.8%)	2 (16.7%)	0.43^1^
Symptom onset to Hospitalisation	7.0 [5.0–9.1]	5.2 [2.7–7.7]	0.09^2^	5.5 [4.0–6.9]	2.4 [0.0–4.8]	**0.04**^2^
BMI	29.9 [28.2–31.7]	31.3 [28.2–34.5]	0.83^2^	28.2 [26.6–29.8]	25.4 [22.3–28.5]	0.20^2^
**Comorbidities**						
Total	32 (97.0%)	25 (96.2%)	0.86^1^	51 (94.4%)	12 (100.0%)	0.40^1^
Arterial hypertension	21 (63.6%)	21 (80.8%)	0.15^1^	37 (68.5%)	11 (91.7%)	0.10^1^
Pre-existing respiratory disease	13 (39.4%)	11 (42.3%)	0.82^1^	24 (44.4%)	6 (50.0%)	0.73^1^
Pre-existing heart disease	10 (30.3%)	14 (53.8%)	0.07^1^	22 (40.7%)	5 (41.7%)	0.95^1^
Overweight (BMI = 25 kg/m2, < 30 kg/m2)	14 (42.4%)	8 (30.8%)	0.36^1^	17 (34.0%)	3 (27.3%)	0.67^1^
Obesity (BMI = 30 kg/m2)	15 (45.5%)	12 (46.2%)	0.96^1^	15 (30.0%)	2 (18.2%)	0.43^1^
Chronic kidney disease	6 (18.2%)	7 (26.9%)	0.42^1^	18 (33.3%)	5 (41.7%)	0.58^1^
Smoking	7 (35.0%)	4 (30.8%)	0.80^1^	20 (64.5%)	3 (50.0%)	0.50^1^
Former smoking	2 (10.0%)	2 (15.4%)	0.64^1^	8 (25.8%)	3 (50.0%)	0.24^1^
Current smoking	5 (25.0%)	2 (15.4%)	0.51^1^	12 (38.7%)	0 (0.0%)	0.06^1^
Diabetes mellitus	4 (12.1%)	9 (34.6%)	**0.04**^1^	15 (27.8%)	3 (27.3%)	0.97^1^
Malignancy	6 (18.2%)	5 (19.2%)	0.92^1^	14 (26.4%)	5 (41.7%)	0.29^1^
Chronic liver failure	0 (0.0%)	0 (0.0%)	-	0 (0.0%)	0 (0.0%)	-
Chronic hepatitis	0 (0.0%)	1 (3.8%)	0.26^1^	5 (9.3%)	0 (0.0%)	0.27^1^
Peripheral arterial occlusive disease	2 (6.1%)	2 (7.7%)	0.80^1^	3 (5.6%)	1 (8.3%)	0.72^1^

Numeric parameters are expressed as median [95%CI], categorical as N(%).

^1^Pearson chi-squared.

^2^Mann-Whitney-U. ARDS = acute respiratory distress syndrome. BMI = body mass index.

Although there was no significant difference in the prevalence of diabetes or prediabetes between subgroups, the BMI levels as a grade of overweight (BMI ≥ 25 kg/m^2^) was significantly higher in ARDS versus non-ARDS patients [28.6 (26.3–31.3) vs. 26.8 (23.9–29.8) kg/m^2^; p = 0.01], but did not differ in survivors versus non-survivors [28.4 (24.7–32.6) vs. 28.6 (24.9–31.0) kg/m^2^; p = 0.59]. Comparing a fatal outcome and survival for the subgroups of ARDS or non ARDS patients showed no significant difference in BMI levels. The mean absolute difference in BMI between ARDS and non-ARDS patients was 1.8 kg/m^2^ (reflecting a difference of about 10 kg between groups) and there was no difference in median BMI between non-survivors and survivors, suggesting that BMI was associated with disease severity, but not with fatal outcome.

### Outcome predictors

**Fig 1:**
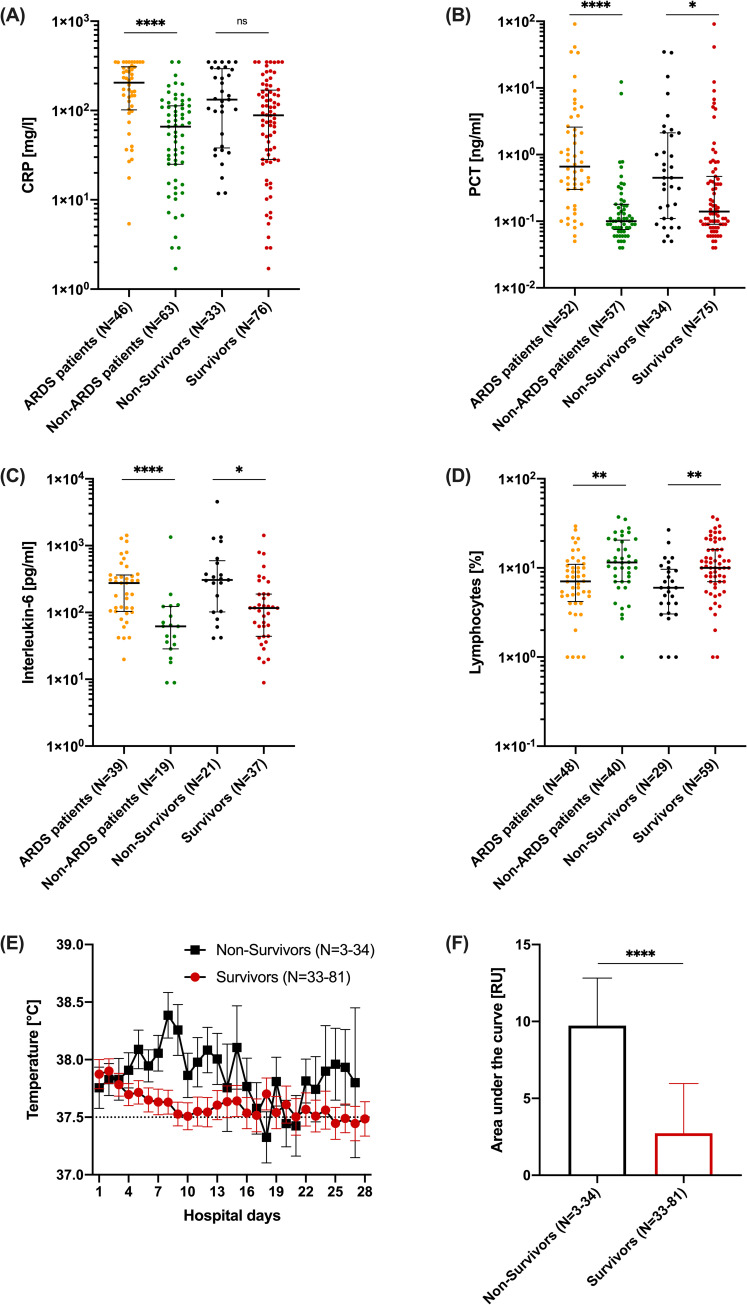
Inflammatory burden compared in the different subgroups. **(A**) CRP (C reactive protein) in mg/l for each individual with Median [IQR25; IQR75] of ARDS patients vs. Non-ARDS patients (orange, 205.3 [101.7; 309.0], N = 46 vs. green, 65.9 [25.0; 113.0], N = 63), Non-Survivors vs. Survivors (black, 132.6 [38.0; 293.5], N = 33 vs. red, 88.4 [28.3; 168.9], N = 76). **(B)** PCT (Procalcitonin) in ng/ml for each individual with Median [IQR25; IQR75] of ARDS patients vs. Non-ARDS patients (orange, 0.66 [0.3; 2.6], N = 52 vs. green, 0.1 [0.07; 0.18], N = 57), Non-Survivors vs Survivors (black, 0.45 [0.11; 2.1], N = 34 vs. red, 0.14 [0.09; 0.47], N = 75). **(C)** Interleukin-6 in pg/ml for each individual with Median [IQR25; IQR75] of ARDS patients vs. Non-ARDS patients (orange, 275.8 [103.4; 364.5], N = 39 vs. green, 61.9 [28.5; 123.0], N = 19), Non-Survivors vs. Survivors (black, 306.8 [102.1; 594.6], N = 21 vs. red, 116.6 [43.9; 188.1], N = 37). **(D)** Lymphocytes in % for each individual with Median [IQR25; IQR75] of ARDS patients vs. Non-ARDS patients (orange, 7.0 [4.2; 10.9], N = 48 vs. green, 11.5 [7.0; 20.5], N = 40), Non-Survivors vs. Survivors (black, 6.0 [3.0; 9.6], N = 29 vs. red, 10.0 [7.0; 16.1], N = 59). **(E)** Mean temperature by hospital days of Non-Survivors (black, Mean ± SEM, N = 3–34) and Survivors (red, Mean ± SEM, N = 33–81). **(F)** Area under the temperature curve (see 1E) in relative arbitrary units with Mean ± SEM for Non-Survivors vs. Survivors (black, 9.7 ± 3.1, N = 3–34 vs. red, 2.7 ± 3.2, N = 33–81).

**Fig 2:**
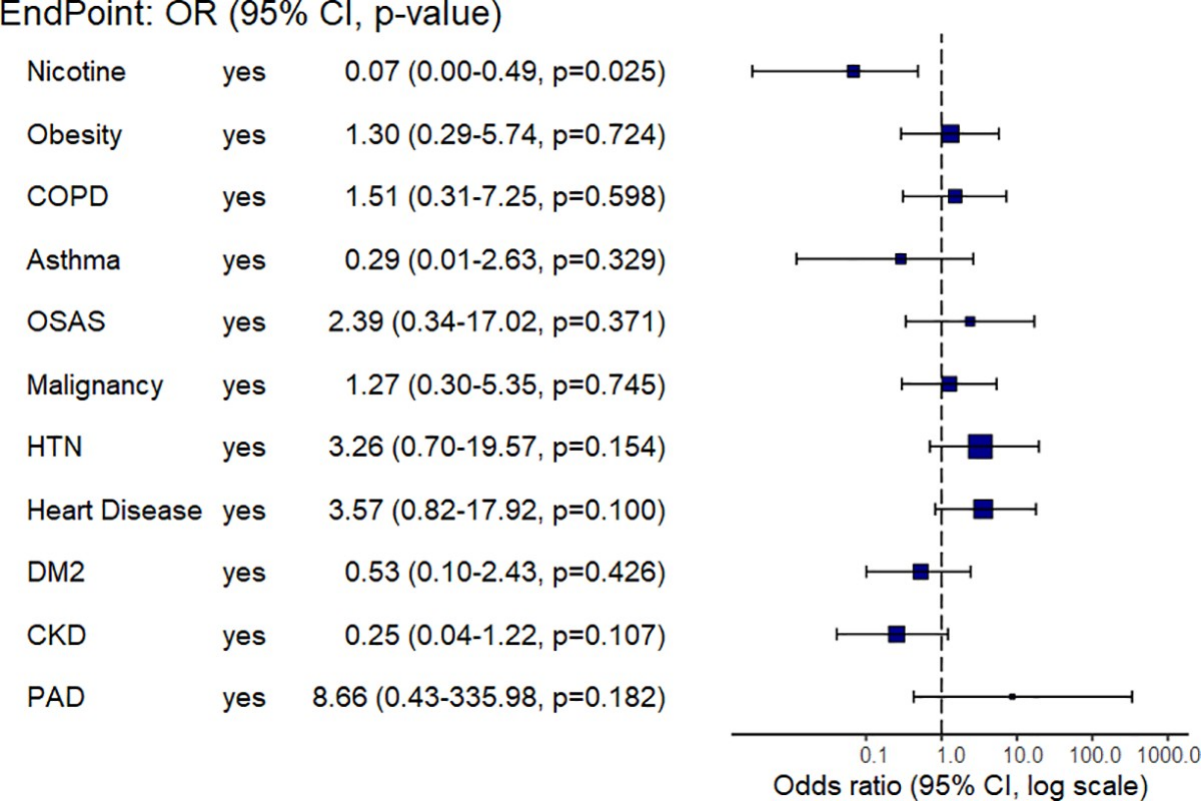
The potential effect of risk factors on patient outcome. Plot of logistic regression with adjusted Odds Ratio (point estimate and 95% CI) of different risk factors (logistic regression by means of base R Generalized Linear Model(glm)). COPD: chronic obstructive lung disease, OSAS: obstructive sleep apnea syndrome, HTN: hypertension, DM2: type 2 diabetes mellitus, CKD: chronic kidney disease, PAD: peripheral artery disease.

Comparing patients with ARDS and non-ARDS, inflammatory parameters like CRP [205.3 (105.2–305.1) vs. 65.9 (25.1–112.8) mg/l; p<0.0001], PCT [0.7 (0.3–2.4) vs. 0.1 (0.1–0.2) ng/ml; p<0.0001], and IL-6 [276.4 (105.1–366.6) vs. 62.1 (33.9–114.3) pg/ml; p = 0.0001] were significantly higher, and lymphocytes [7.1 (4.6–10.7) vs. 11.5 (7.0–19.9)%; p = 0.003] were significantly lower, at the time of hospital admission (Fig 1). Comparing non-survivors with survivors, CRP [132.6 (39.6–287.2) vs. 88.5 (28.4–63.6) mg/l; p = 0.05], PCT [0.5 (0.1–2.1) vs. 0.1 (0.1–0.5) ng/ml; p = 0.02], IL-6 [306.8 (103.4–547.6) vs. 116.6 (44.2–186.4) pg/ml; p = 0.02] and lymphocytes [6.0 (3.1–9.3) vs. 10.0 (7.0–16.1)%; p = 0.001] were numerically or even significantly different (Fig 1). D-Dimers, as coagulation parameter, showed no significance between survivors and non-survivors [3421 (1347–12046) vs. 1418 (956.5–5768.5) ng/ml; p = 0.90], but in non ARDS patients D-Dimers in non-survivors were significantly higher [1042.6 [683.8–1,401.5] vs. 10753.3 [-24120.8–45627.5]; p = 0.01]. In ARDS patients also compared to non ARDS patients D-Dimers levels were significantly higher [3496.5 (1394.8–8452.0) vs. 763.5 (613.3–939.0) ng/ml; p = 0.001]. LDH [435 (346.5–550.3) vs. 332 (263.3–434.3) U/l] and urea [58 (34.0–80.5) vs. 39 (28.0–58.8) mg/dl] were significantly higher in ARDS versus non-ARDS patients (both p<0.05). In addition, LDH [433 (332–544) vs. 369 (292.5–447.5) U/l; p = 0.45] and urea [53 (41–87) vs. 39 (27.8–67) mg/dl; p = 0.2] were different comparing non-survivors versus survivors. In the uni- and multivariate logistic regressions concerning predictive parameters a significance for smoking was observed (odds ratio: 0.07 [0.0–0.49], p = 0.025), there was no significance observed for other parameters like obesity, COPD, Asthma bronchiale, obstructive sleep apnoea syndrome (OSAS), malignancy, hypertension, heart disease, diabetes mellitus, chronic kidney disease and PAD (Fig 2).

**Fig 3:**
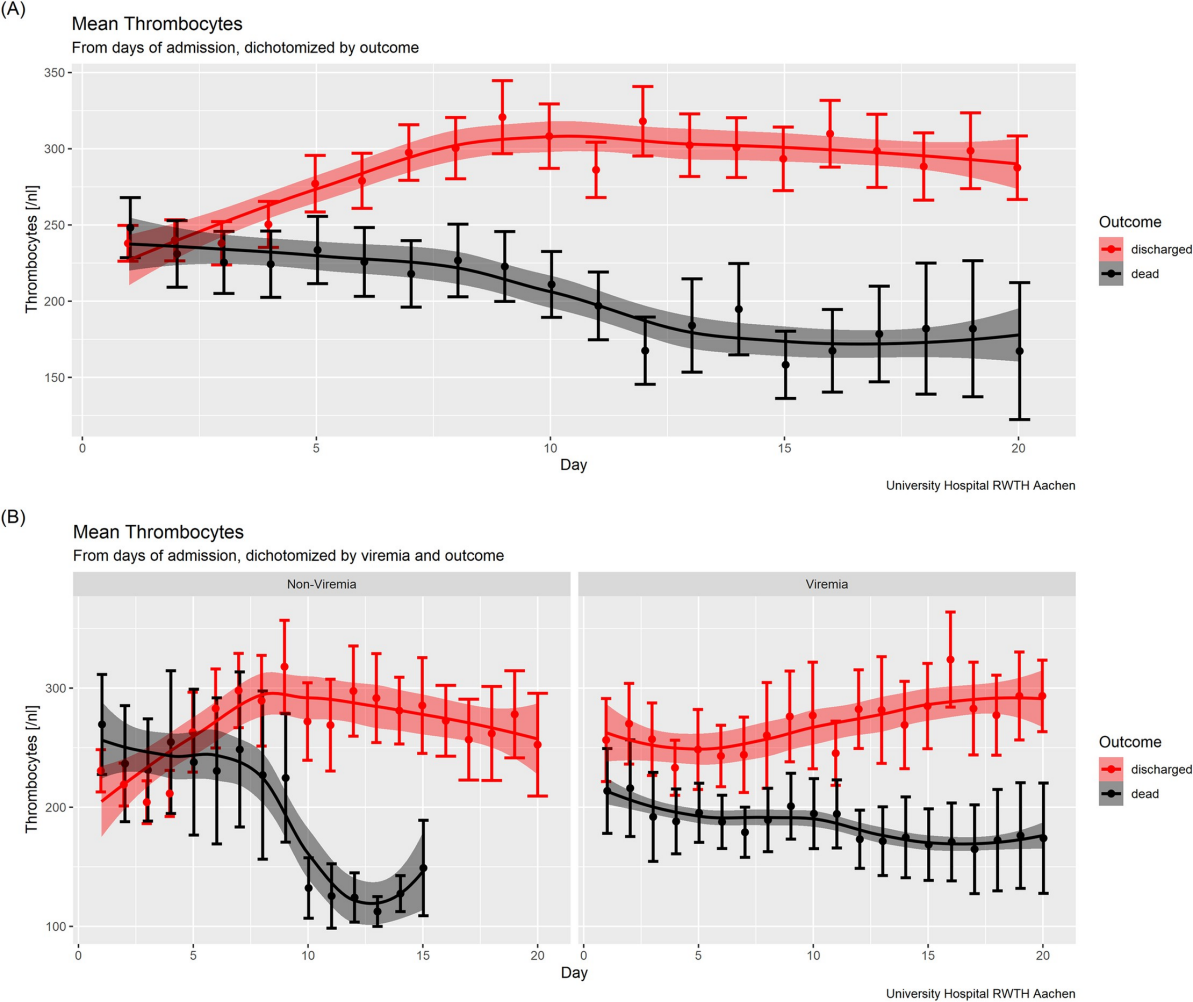
The potential impact on viremia on patient outcome via thrombocyte course. **(A)** Thrombocytes in 1/nl by hospital days for Non-Survivors (N = 4–28) and Survivors (N = 9–57). **(B)** Thrombocytes in 1/nl by hospital days dichotomized by viremia (sero-positive on the left-hand side vs. sero-negative on the right-hand side) for Non-Survivors (sero-positive: N = 4–7, sero-negative: N = 4–5) and Survivors (sero-positive N = 4–7, sero-negative N = 4–23). Patient numbers vary between different time points in the figures, but the time point refers to the initial admission for each patient.

Since an ongoing inflammatory reaction or „storm”has been discussed as a denominator for clinical outcome [9, 10], we analysed temperature curves as an easy to assess clinical parameter of inflammation in this context. To this end, we calculated the respective area under the fever curve in relative arbitrary units in relation to 37.5°C, reflecting the “load” of fever (Fig 1E and 1F). The comparison between survivors and non-survivors showed a marked and significant difference in fever load (a 3.5-fold increase in relative arbitrary units; p<0.0001) between these two groups, as a clinical indicator of inflammation (Fig 1F). With respect to viral load (absolute copy number), there were no significant differences between non-survivors and survivors, but an extrapulmonary manifestation of SARS-CoV-2 in non-survivors was detected significantly more often than in survivors (69% vs. 27%; p = 0.002) (Table 3). In addition, detection of SARS-CoV-2 in serum at admission was associated with a significantly increased risk of death (60% vs. 29%; p = 0.01) and a significantly more severe clinical course (60% vs. 17%, p = 0.0002) (Table 3). Interestingly, when patients were dichotomized according to SARS-CoV-2 viremia, other potential risk indicators, such as platelet count over time, showed a marked difference between non-survivors and survivors, suggesting that viremia has detrimental effects via various mechanisms (Fig 3).

**Table 3:** Microbiological findings of the different subgroups.

	N/N total (%)				N/N total (%)		
	All patients (N = 125)	ARDS patients (N = 59)	non-ARDS patients (N = 66)	P values	Non-Survivors (N = 38)	Survivors (N = 87)	P values
**Viral load**							
High	22/113 (19)	10/52 (19)	12/61 (20)	>0.99	7/37 (19)	15/76 (20)	>0.99
Medium	66/113 (58)	32/52 (62)	34/61 (56)	0.57	24/37 (65)	42/76 (55)	0.54
Low	25/113 (22)	10/52 (19)	15/61 (25)	0.64	6/37 (16)	19/76 (25)	0.33
Ct S-Gen—Median (IQR)	25.6 (20.7–29.4)	25.6 (20.7–29.3)	25.2 (20.2–29.7)	0.60	25,5 (20,8–29,4)	25,6 (20,6–29,4)	0.88
**Viral detection**							
Pulmonary manifestation	107/123 (87)	46/58 (79)	61/65 (94)	**0.02**	34/38 (89)	73/85 (86)	0.77
positive out of hospital	18 (14)	11 (31)	7 (27)	-	3 (8)	15 (17)	-
Extrapulmonary manifestation	40/95 (42)	27/53 (51)	13/42 (31)	0.06	18/26 (69)	18/66 (27)	**0.002**
Serum	27/71 (38)	21/35 (60)	6/36 (17)	**0.0002**	12/20 (60)	15/51 (29)	**0.02**
Stool	13/40 (33)	6/20 (30)	7/20 (35)	>0.99	6/10 (60)	7/30 (23)	0.05
Urine	12/63 (19)	7/36 (19)	5/27 (19)	>0.99	7/20 (35)	5/43 (12)	**0.03**
**Bacterial detection**							
Blood culture	24/109 (22)	19/58 (33)	5/51 (10)	**0.005**	10/36 (28)	15/73 (21)	0.46
Urine culture	47/99 (47)	26/53 (49)	21/46 (46)	0.84	13/31 (42)	35/69 (51)	0.51

Data in N/N total (%) or Median (IQR). In ordinal scaled parameters testing for normal distribution and calculation of P values with Welch test or Wilcoxon-Mann-Whitney test. P values of nominal scaled parameter calculated with Fisher’s exact test. SEM = standard error of the mean. ARDS = acute respiratory distress syndrome. IQR = interquartile range. Ct = cyclic threshold.

**Fig 4:**
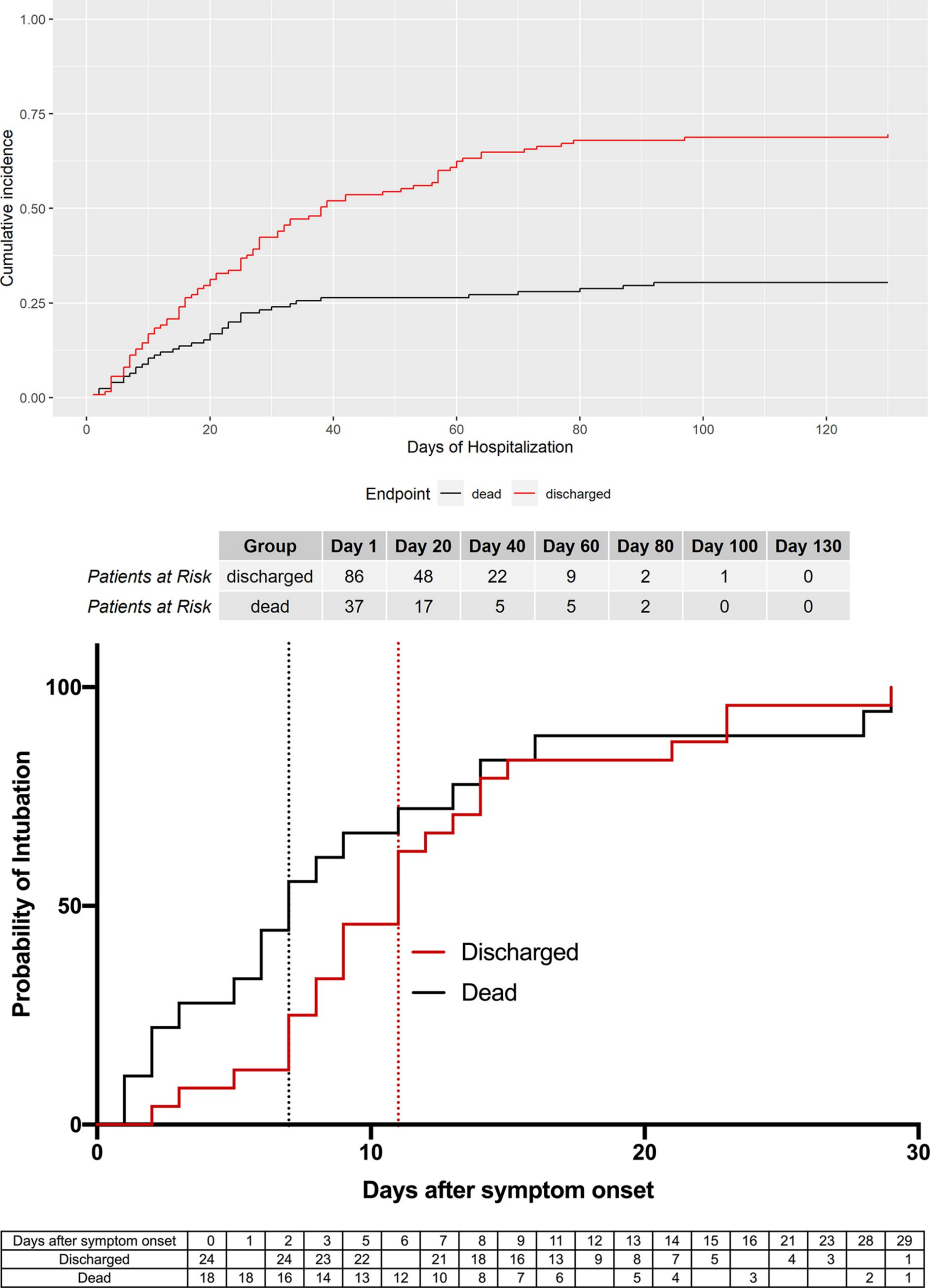
The probability of survival and intubation for patient outcome. **(A)** Cumulative incidence (Dead: N = 38; Discharged: N = 87; no censored patients). **(B)** Probability of intubation of all ARDS patients, discharged vs. dead, with Kaplan-Meier method (dead: N = 24, 2 censored patients; discharged: N = 18).

Cumulative incidence analysis suggests that 50% of all non-survivors died within the first 20 days after the onset of symptoms (Fig 4A). In the subgroup of ARDS patients, 50% of all non-survivors required intubation and invasive ventilation within the first 7 days of onset of symptoms. In contrast, if there was no need for mechanical ventilation within 16 days of symptom onset, 90% of patients survived (Fig 4B).

## Discussion

This report of 125 patients hospitalized due to COVID-19 infection in Germany, is showing an increased inflammatory burden and SARS-CoV-2 seropositivity as early markers in patients with ARDS or for fatal outcome.

In different reports of large cohorts, mainly from China, Italy, or the US, comorbidities like pulmonary and cardiovascular diseases, as well as diabetes and kidney diseases, have been proposed as risk factors, or were associated with worse outcomes [5, 6, 11, 12]. Interestingly, in our study, there were no highly significant differences in comorbidities between subgroups, although all patients had comorbidities; neither in ARDS versus non-ARDS patients, nor in non-survivors versus survivors. This also applied for renin-angiotensin-aldosterone system (RAAS)-inhibitor medication, as was recently reported by others [13, 14]. In our cohort, overweight and higher BMI was associated with severe clinical course of disease but not with fatal outcome. This has not been reported previously, and might be due to most reports coming from asian populations with a lower degree of overweight in general [15, 16]. However, the prevalence of known or unknown diabetes and prediabetes were comparable between patients with and without ARDS, and survivors and non-survivors. Additionally, there were no significant differences in HbA1c levels, although a trend might exist. This must be further evaluated in larger populations, as chronic elevated blood glucose levels may trigger an inflammatory response and increased susceptibility to endothelium damage, which has been described post-mortem as a characteristic feature of COVID-19 [12, 17, 18]. The most prominent difference in survival between the subgroups, was time between symptom onset and hospitalization, with patients who died having significantly less time between symptom onset and hospitalization. Age was different in both investigated subgroups, confirming other data that increasing age is associated with more progressive disease and fatal outcomes [5, 7].

In order to identify discriminating early markers for severity and disease outcome at hospital admission, we observed significant differences in inflammatory markers. An increased inflammatory reaction, or so called „cytokine storm“, has been previously described [9, 19], and our data suggest that an early increase in these markers is associated with poor prognosis. To further analyse the ongoing inflammatory burden, we used fever as an easy to assess clinical indicator. The calculated area under the fever curve (fever load) was significantly different between survivors and non-survivors, supporting the concept of a higher inflammatory response and burden in patients with severe outcomes. In addition, elevated levels of urea in patients at admission were associated with ARDS or fatal outcome, indicating a pronounced catabolic state early on in the disease. Despite some reports and observations about peculiar alterations in coagulation and thrombembolic events [20], we didn´t find any significant differences in D-Dimer, INR or PTT at time of admission between survivors and non-survivors, but D-Dimer were significantly higher in patients with than without ARDS.

Viral load dynamics in relation to disease severity has been reported recently [21]. Overall, in our cohort viral load was comparable between all subgroups, but additional analyses revealed that extrapulmonary SARS-CoV-2 detection, and especially viremia, was associated with more severe disease and fatal outcome. Given this, it is worth mentioning that thrombocytopenia has been reported in COVID-19 patients with poor outcomes [22–24]. In our study, the number of thrombocytes were not different between subgroups on admission. However, in patients with viremia—but not in those with SARS-CoV-2 RNA negative blood—thrombocyte levels significantly diverged between survivors and non-survivors throughout the course of disease. This might explain the difference to the studies cited above; the thrombocyte count timing is important when comparing studies.

It didn´t escape our attention that this is the first data of a tertiary care center in Germany, which is currently able to provide sufficient intensive care to patients with COVID-19. At the same time, the rather specific cohort of patients treated in our university hospital is the major limitation of this study, the evidence and scientific contribution is rather descriptive. Furthermore, we are aware that some significant associations might be due to small numbers or lack of multiple testing, for which we didn´t adjust. However, we hope that the results and conclusions will incite further evaluation in different ongoing studies worldwide.

### Study limitations

This study has several limitations. First, the character of retrospectively collected data limited the completeness of the data and made missing data unavoidable. Second, recruitment of the patients differed in the disease stage including patients with and without ARDS concerning the onset of the disease. Some patients were transferred to our hospital from other ICUs already diagnosed with ARDS. Third, Given the comparable small number of patients and events further analyses including logistic regressions are limited in their potential to identify significant relations.

In conclusion, we have identified early risk markers for a severe clinical course like ARDS or fatal outcome in patients being hospitalized for COVID-19. Simple laboratory markers in addition to SARS-CoV-2 viremia, age and time of symptom onset to hospitalization seems to be feasible in predicting survival.

## References

[1] Wu C, Chen X, Cai Y, Xia J, Zhou X, Xu S, et al Risk Factors Associated With Acute Respiratory Distress Syndrome and Death in Patients With Coronavirus Disease 2019 Pneumonia in Wuhan, China. JAMA Intern Med. 2020 10.1001/jamainternmed.2020.0994 32167524 PMC7070509

[2] Wang D, Hu B, Hu C, Zhu F, Liu X, Zhang J, et al Clinical Characteristics of 138 Hospitalized Patients With 2019 Novel Coronavirus-Infected Pneumonia in Wuhan, China. JAMA. 2020 10.1001/jama.2020.1585 32031570 PMC7042881

[3] Rodriguez-Morales AJ, Cardona-Ospina JA, Gutierrez-Ocampo E, Villamizar-Pena R, Holguin-Rivera Y, Escalera-Antezana JP, et al Clinical, laboratory and imaging features of COVID-19: A systematic review and meta-analysis. Travel Med Infect Dis. 2020:101623 10.1016/j.tmaid.2020.101623 32179124 PMC7102608

[4] Mehra MR, Desai SS, Kuy S, Henry TD, Patel AN. Cardiovascular Disease, Drug Therapy, and Mortality in Covid-19. N Engl J Med. 2020. 10.1056/NEJMoa2007621 PMC7206931 32356626

[5] Richardson S, Hirsch JS, Narasimhan M, Crawford JM, McGinn T, Davidson KW, et al Presenting Characteristics, Comorbidities, and Outcomes Among 5700 Patients Hospitalized With COVID-19 in the New York City Area. JAMA. 2020. 10.1001/jama.2020.6775 PMC7177629 32320003

[6] Grasselli G, Zangrillo A, Zanella A, Antonelli M, Cabrini L, Castelli A, et al Baseline Characteristics and Outcomes of 1591 Patients Infected With SARS-CoV-2 Admitted to ICUs of the Lombardy Region, Italy. JAMA. 2020 10.1001/jama.2020.5394 32250385 PMC7136855

[7] Zhou F, Yu T, Du R, Fan G, Liu Y, Liu Z, et al Clinical course and risk factors for mortality of adult inpatients with COVID-19 in Wuhan, China: a retrospective cohort study. The Lancet. 10.1016/S0140-6736(20)30566-3 32171076 PMC7270627

[8] Dreher M, Kersten A, Bickenbach J, Balfanz P, Hartmann B, Cornelissen C, et al The characteristics of 50 hospitalized COVID-19 patients with and without ARDS. Deutsches Aerzteblatt Online. 2020 10.3238/arztebl.2020.0271 32519944 PMC7171478

[9] Jose RJ, Manuel A. COVID-19 cytokine storm: the interplay between inflammation and coagulation. The Lancet Respiratory Medicine. 2020 10.1016/S2213-2600(20)30216-2 32353251 PMC7185942

[10] Moore JB, June CH. Cytokine release syndrome in severe COVID-19. Science. 2020;368(6490):473–4. 10.1126/science.abb8925 32303591

[11] Cheng Y, Luo R, Wang K, Zhang M, Wang Z, Dong L, et al Kidney disease is associated with in-hospital death of patients with COVID-19. Kidney Int. 2020;97(5):829–38. 10.1016/j.kint.2020.03.005 32247631 PMC7110296

[12] Ma RCW, Holt RIG. COVID-19 and diabetes. Diabet Med. 2020;37(5):723–5. 10.1111/dme.14300 32242990 PMC7228343

[13] Mancia G, Rea F, Ludergnani M, Apolone G, Corrao G. Renin-Angiotensin-Aldosterone System Blockers and the Risk of Covid-19. N Engl J Med. 2020 10.1056/NEJMoa2006923 32356627 PMC7206933

[14] Patel AB, Verma A. COVID-19 and Angiotensin-Converting Enzyme Inhibitors and Angiotensin Receptor Blockers: What Is the Evidence? JAMA. 2020 10.1001/jama.2020.4812 32208485

[15] Kass DA, Duggal P, Cingolani O. Obesity could shift severe COVID-19 disease to younger ages. The Lancet. 10.1016/S0140-6736(20)31024-2 32380044 PMC7196905

[16] Dietz W, Santos-Burgoa C. Obesity and its Implications for COVID-19 Mortality. Obesity.n/a(n/a). 10.1002/oby.22818 32237206

[17] Varga Z, Flammer AJ, Steiger P, Haberecker M, Andermatt R, Zinkernagel AS, et al Endothelial cell infection and endotheliitis in COVID-19. The Lancet. 2020;395(10234):1417–8. 10.1016/S0140-6736(20)30937-5 32325026 PMC7172722

[18] Bornstein SR, Rubino F, Khunti K, Mingrone G, Hopkins D, Birkenfeld AL, et al Practical recommendations for the management of diabetes in patients with COVID-19. The Lancet Diabetes & Endocrinology. 2020 10.1016/S2213-8587(20)30152-2 32334646 PMC7180013

[19] Zhang C, Wu Z, Li JW, Zhao H, Wang GQ. The cytokine release syndrome (CRS) of severe COVID-19 and Interleukin-6 receptor (IL-6R) antagonist Tocilizumab may be the key to reduce the mortality. Int J Antimicrob Agents. 2020:105954 10.1016/j.ijantimicag.2020.105954 32234467 PMC7118634

[20] Tang N, Bai H, Chen X, Gong J, Li D, Sun Z. Anticoagulant treatment is associated with decreased mortality in severe coronavirus disease 2019 patients with coagulopathy. J Thromb Haemost. 2020;18(5):1094–9. 10.1111/jth.14817 32220112 PMC9906401

[21] Zheng S, Fan J, Yu F, Feng B, Lou B, Zou Q, et al Viral load dynamics and disease severity in patients infected with SARS-CoV-2 in Zhejiang province, China, January-March 2020: retrospective cohort study. BMJ. 2020;369:m1443 10.1136/bmj.m1443 32317267 PMC7190077

[22] Zhang Y, Xiao M, Zhang S, Xia P, Cao W, Jiang W, et al Coagulopathy and Antiphospholipid Antibodies in Patients with Covid-19. New England Journal of Medicine. 2020;382(17):e38 10.1056/NEJMc2007575 32268022 PMC7161262

[23] Oxley TJ, Mocco J, Majidi S, Kellner CP, Shoirah H, Singh IP, et al Large-Vessel Stroke as a Presenting Feature of Covid-19 in the Young. New England Journal of Medicine. 2020:e60. 10.1056/NEJMc2009787 PMC7207073 32343504

[24] Yang X, Yang Q, Wang Y, Wu Y, Xu J, Yu Y, et al Thrombocytopenia and its association with mortality in patients with COVID-19. Journal of Thrombosis and Haemostasis.n/a(n/a). 10.1111/jth.14848 32302435 PMC9906135

